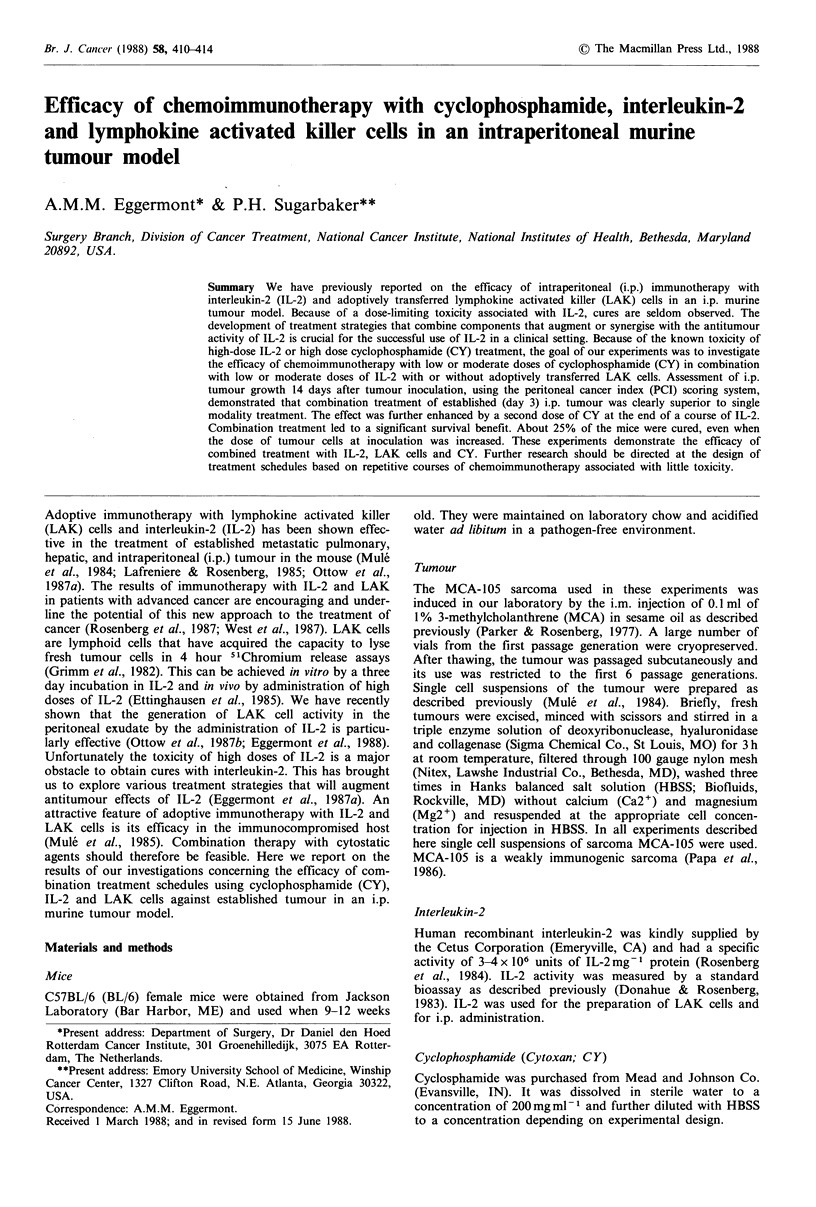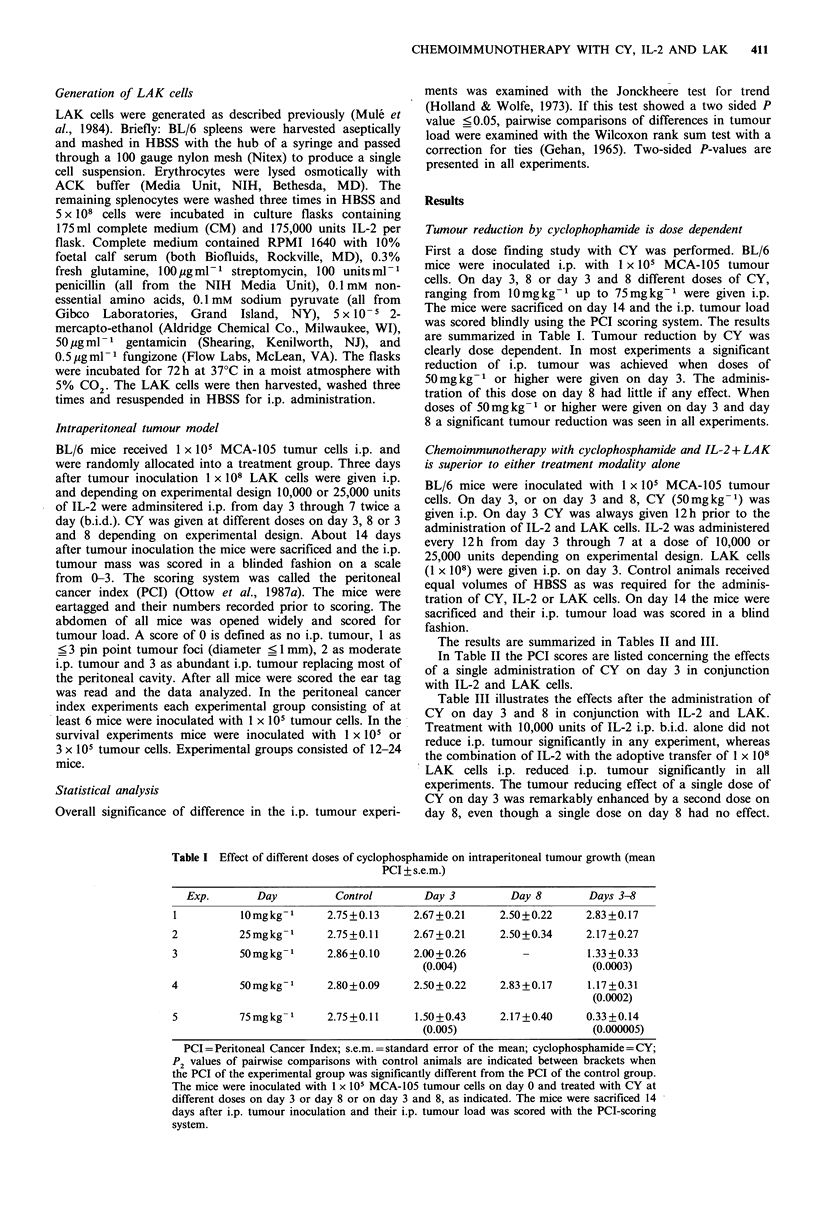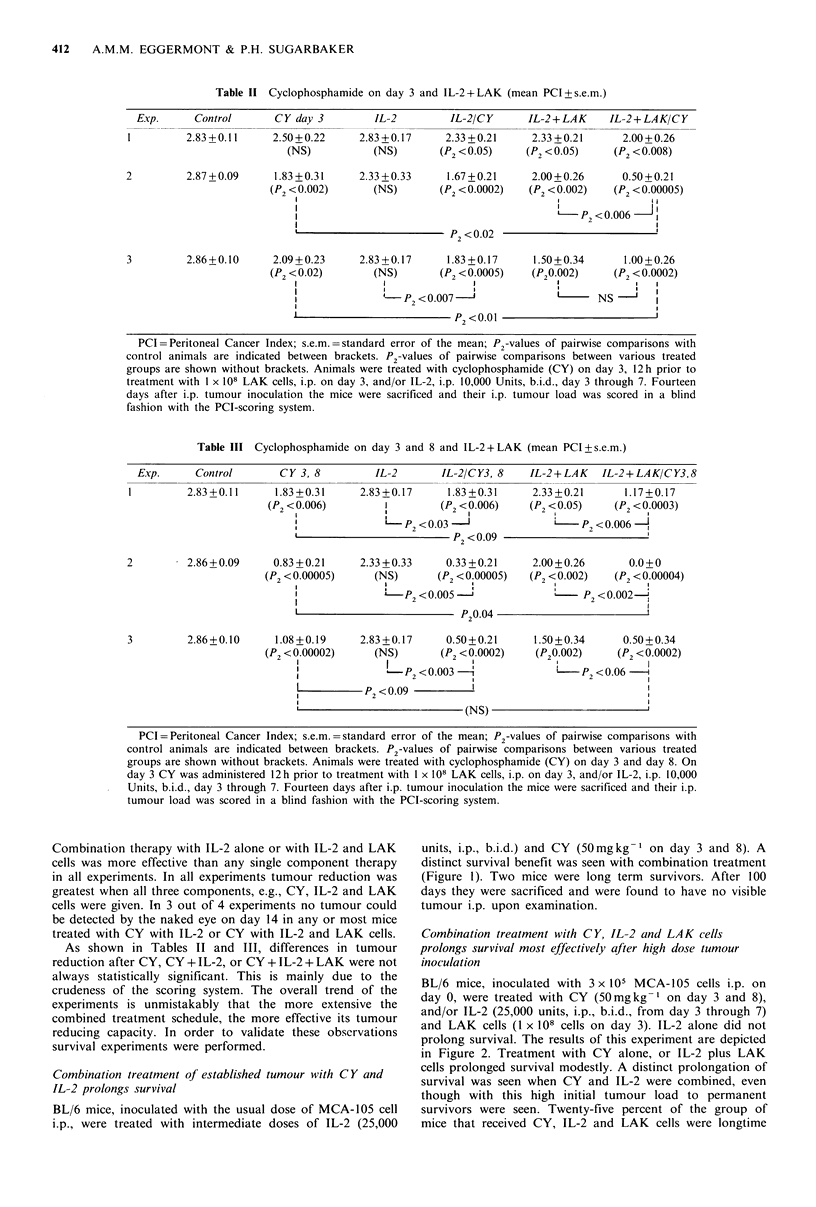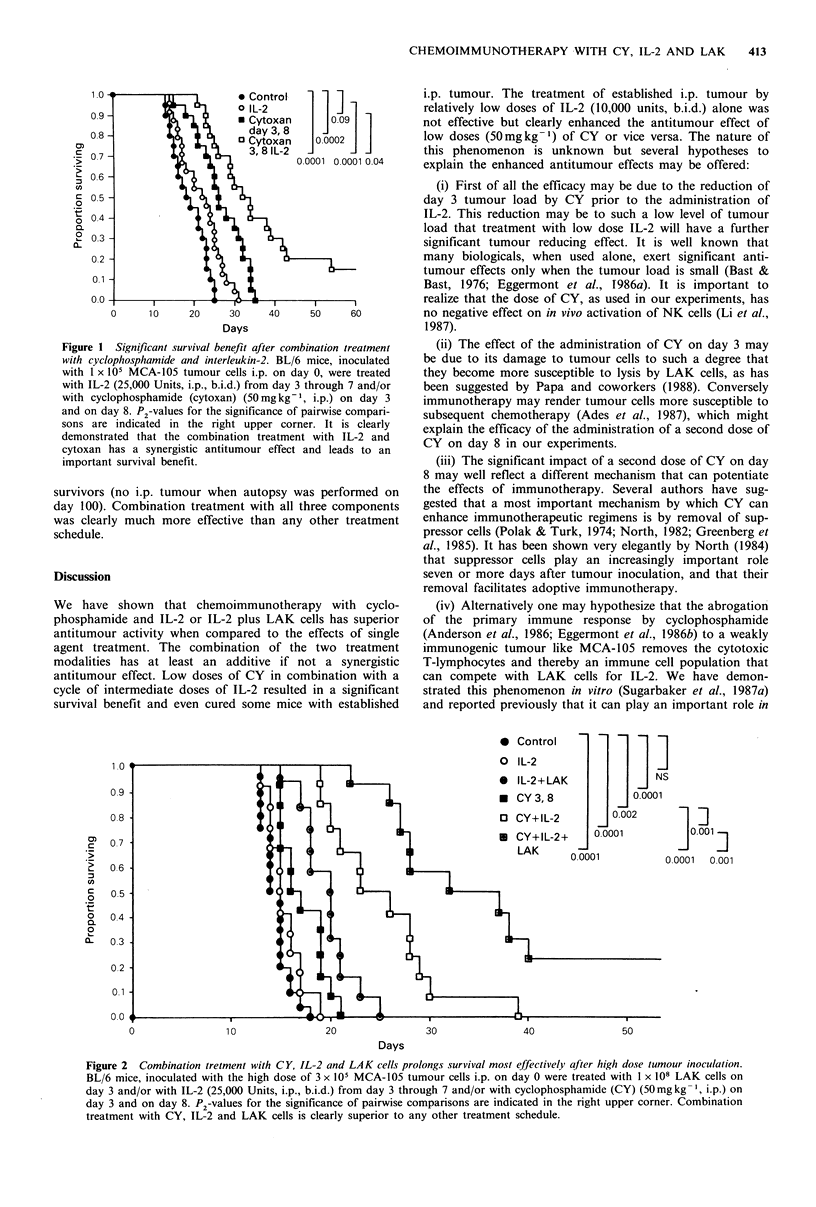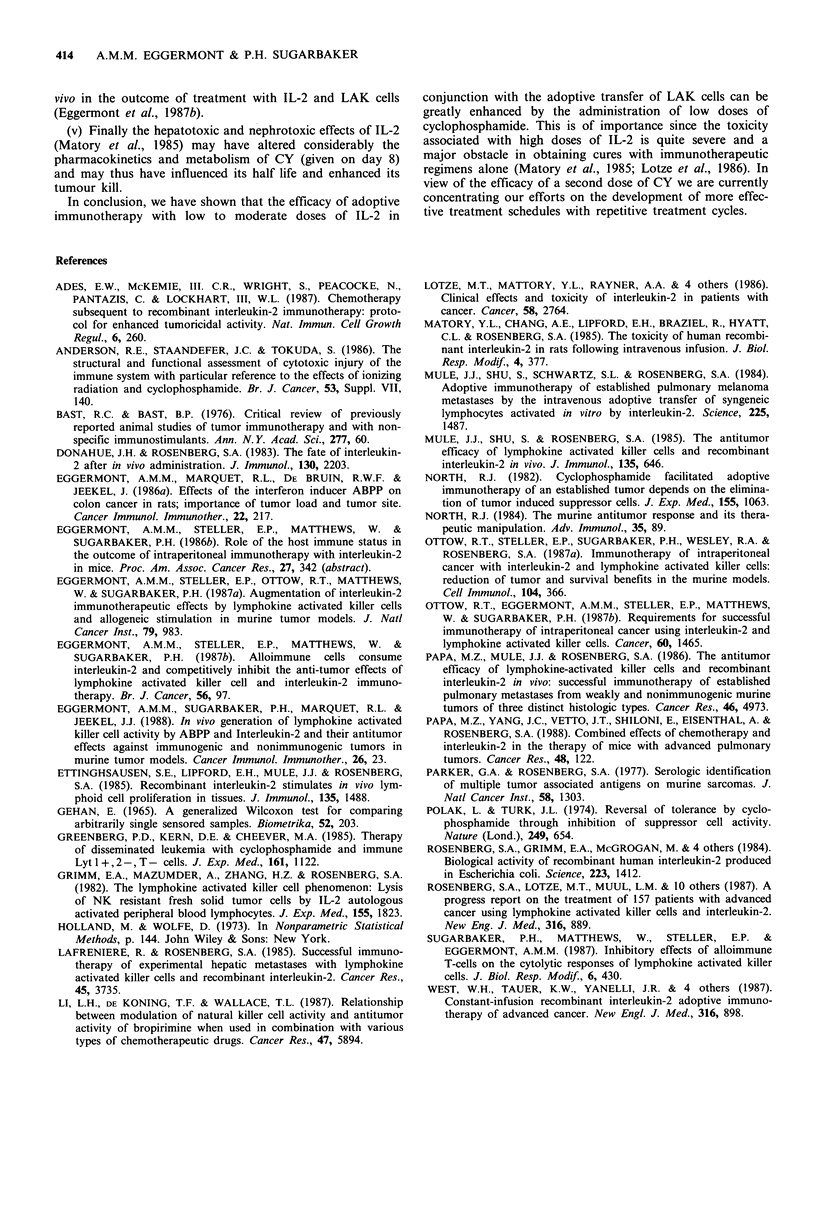# Efficacy of chemoimmunotherapy with cyclophosphamide, interleukin-2 and lymphokine activated killer cells in an intraperitoneal murine tumour model.

**DOI:** 10.1038/bjc.1988.231

**Published:** 1988-10

**Authors:** A. M. Eggermont, P. H. Sugarbaker

**Affiliations:** Division of Cancer Treatment, National Cancer Institute, Bethesda, Maryland 20892.

## Abstract

We have previously reported on the efficacy of intraperitoneal (i.p.) immunotherapy with interleukin-2 (IL-2) and adoptively transferred lymphokine activated killer (LAK) cells in an i.p. murine tumour model. Because of a dose-limiting toxicity associated with IL-2, cures are seldom observed. The development of treatment strategies that combine components that augment or synergise with the antitumour activity of IL-2 is crucial for the successful use of IL-2 in a clinical setting. Because of the known toxicity of high-dose IL-2 or high dose cyclophosphamide (CY) treatment, the goal of our experiments was to investigate the efficacy of chemoimmunotherapy with low or moderate doses of cyclophosphamide (CY) in combination with low or moderate doses of IL-2 with or without adoptively transferred LAK cells. Assessment of i.p. tumour growth 14 days after tumour inoculation, using the peritoneal cancer index (PCI) scoring system, demonstrated that combination treatment of established (day 3) i.p. tumour was clearly superior to single modality treatment. The effect was further enhanced by a second dose of CY at the end of a course of IL-2. Combination treatment led to a significant survival benefit. About 25% of the mice were cured, even when the dose of tumour cells at inoculation was increased. These experiments demonstrate the efficacy of combined treatment with IL-2, LAK cells and CY. Further research should be directed at the design of treatment schedules based on repetitive courses of chemoimmunotherapy associated with little toxicity.


*Br. J. Cancer* (1988) **58**, 410–414 © The Macmillan Press Ltd., 1988

### Efficacy of chemoimmunotherapy with cyclophosphamide, interleukin-2 and lymphokine activated killer cells in an intraperitoneal murine tumour model

A.M.M. Eggermont* & P.H. Sugarbaker**

*Surgery Branch, Division of Cancer Treatment, National Cancer Institute, National Institutes of Health, Bethesda, Maryland 20892, USA.*

**Summary** We have previously reported on the efficacy of intraperitoneal (i.p.) immunotherapy with interleukin-2 (IL-2) and adoptively transferred lymphokine activated killer (LAK) cells in an i.p. murine tumour model. Because of a dose-limiting toxicity associated with IL-2, cures are seldom observed. The development of treatment strategies that combine components that augment or synergise with the antitumour activity of IL-2 is crucial for the successful use of IL-2 in a clinical setting. Because of the known toxicity of high-dose IL-2 or high dose cyclophosphamide (CY) treatment, the goal of our experiments was to investigate the efficacy of chemoimmunotherapy with low or moderate doses of cyclophosphamide (CY) in combination with low or moderate doses of IL-2 with or without adoptively transferred LAK cells. Assessment of i.p. tumour growth 14 days after tumour inoculation, using the peritoneal cancer index (PCI) scoring system, demonstrated that combination treatment of established (day 3) i.p. tumour was clearly superior to single modality treatment. The effect was further enhanced by a second dose of CY at the end of a course of IL-2. Combination treatment led to a significant survival benefit. About 25% of the mice were cured, even when the dose of tumour cells at inoculation was increased. These experiments demonstrate the efficacy of combined treatment with IL-2, LAK cells and CY. Further research should be directed at the design of treatment schedules based on repetitive courses of chemoimmunotherapy associated with little toxicity.

Adoptive immunotherapy with lymphokine activated killer (LAK) cells and interleukin-2 (IL-2) has been shown effective in the treatment of established metastatic pulmonary, hepatic, and intraperitoneal (i.p.) tumour in the mouse (Mulé *et al.*, 1984; Lafreniere & Rosenberg, 1985; Ottow *et al.*, 1987*a*). The results of immunotherapy with IL-2 and LAK in patients with advanced cancer are encouraging and underline the potential of this new approach to the treatment of cancer (Rosenberg *et al.*, 1987; West *et al.*, 1987). LAK cells are lymphoid cells that have acquired the capacity to lyse fresh tumour cells in 4 hour $^{51}$Chromium release assays (Grimm *et al.*, 1982). This can be achieved *in vitro* by a three day incubation in IL-2 and *in vivo* by administration of high doses of IL-2 (Ettinghausen *et al.*, 1985). We have recently shown that the generation of LAK cell activity in the peritoneal exudate by the administration of IL-2 is particularly effective (Ottow *et al.*, 1987*b*; Eggermont *et al.*, 1988). Unfortunately the toxicity of high doses of IL-2 is a major obstacle to obtain cures with interleukin-2. This has brought us to explore various treatment strategies that will augment antitumour effects of IL-2 (Eggermont *et al.*, 1987*a*). An attractive feature of adoptive immunotherapy with IL-2 and LAK cells is its efficacy in the immunocompromised host (Mulé *et al.*, 1985). Combination therapy with cytostatic agents should therefore be feasible. Here we report on the results of our investigations concerning the efficacy of combination treatment schedules using cyclophosphamide (CY), IL-2 and LAK cells against established tumour in an i.p. murine tumour model.

### Materials and methods

### *Mice*

C57BL/6 (BL/6) female mice were obtained from Jackson Laboratory (Bar Harbor, ME) and used when 9–12 weeks old. They were maintained on laboratory chow and acidified water *ad libitum* in a pathogen-free environment.

### *Tumour*

The MCA-105 sarcoma used in these experiments was induced in our laboratory by the i.m. injection of 0.1 ml of 1% 3-methylcholanthrene (MCA) in sesame oil as described previously (Parker & Rosenberg, 1977). A large number of vials from the first passage generation were cryopreserved. After thawing, the tumour was passaged subcutaneously and its use was restricted to the first 6 passage generations. Single cell suspensions of the tumour were prepared as described previously (Mulé *et al.*, 1984). Briefly, fresh tumours were excised, minced with scissors and stirred in a triple enzyme solution of deoxyribonuclease, hyaluronidase and collagenase (Sigma Chemical Co., St Louis, MO) for 3 h at room temperature, filtered through 100 gauge nylon mesh (Nitex, Lawshe Industrial Co., Bethesda, MD), washed three times in Hanks balanced salt solution (HBSS; Biofluids, Rockville, MD) without calcium ($Ca2^{+}$) and magnesium ($Mg2^{+}$) and resuspended at the appropriate cell concentration for injection in HBSS. In all experiments described here single cell suspensions of sarcoma MCA-105 were used. MCA-105 is a weakly immunogenic sarcoma (Papa *et al.*, 1986).

### *Interleukin-2*

Human recombinant interleukin-2 was kindly supplied by the Cetus Corporation (Emeryville, CA) and had a specific activity of $3–4 \times 10^6$ units of IL-2 $mg^{-1}$ protein (Rosenberg *et al.*, 1984). IL-2 activity was measured by a standard bioassay as described previously (Donahue & Rosenberg, 1983). IL-2 was used for the preparation of LAK cells and for i.p. administration.

### *Cyclophosphamide (Cytoxan; CY)*

Cyclosphamide was purchased from Mead and Johnson Co. (Evansville, IN). It was dissolved in sterile water to a concentration of 200 mg $ml^{-1}$ and further diluted with HBSS to a concentration depending on experimental design.

*Present address: Department of Surgery, Dr Daniel den Hoed Rotterdam Cancer Institute, 301 Groenehilledijk, 3075 EA Rotterdam, The Netherlands.

**Present address: Emory University School of Medicine, Winship Cancer Center, 1327 Clifton Road, N.E. Atlanta, Georgia 30322, USA.

Correspondence: A.M.M. Eggermont.

Received 1 March 1988; and in revised form 15 June 1988.

*Generation of LAK cells*

LAK cells were generated as described previously (Mulé *et al.*, 1984). Briefly: BL/6 spleens were harvested aseptically and mashed in HBSS with the hub of a syringe and passed through a 100 gauge nylon mesh (Nitex) to produce a single cell suspension. Erythrocytes were lysed osmotically with ACK buffer (Media Unit, NIH, Bethesda, MD). The remaining splenocytes were washed three times in HBSS and $5 \times 10^8$ cells were incubated in culture flasks containing 175 ml complete medium (CM) and 175,000 units IL-2 per flask. Complete medium contained RPMI 1640 with 10% foetal calf serum (both Biofluids, Rockville, MD), 0.3% fresh glutamine, 100 $\mu$g ml$^{-1}$ streptomycin, 100 units ml$^{-1}$ penicillin (all from the NIH Media Unit), 0.1 mM non-essential amino acids, 0.1 mM sodium pyruvate (all from Gibco Laboratories, Grand Island, NY), $5 \times 10^{-5}$ 2-mercapto-ethanol (Aldridge Chemical Co., Milwaukee, WI), 50 $\mu$g ml$^{-1}$ gentamicin (Shearing, Kenilworth, NJ), and 0.5 $\mu$g ml$^{-1}$ fungizone (Flow Labs, McLean, VA). The flasks were incubated for 72 h at 37°C in a moist atmosphere with 5% $CO_2$. The LAK cells were then harvested, washed three times and resuspended in HBSS for i.p. administration.

*Intraperitoneal tumour model*

BL/6 mice received $1 \times 10^5$ MCA-105 tumur cells i.p. and were randomly allocated into a treatment group. Three days after tumour inoculation $1 \times 10^8$ LAK cells were given i.p. and depending on experimental design 10,000 or 25,000 units of IL-2 were adminsitered i.p. from day 3 through 7 twice a day (b.i.d.). CY was given at different doses on day 3, 8 or 3 and 8 depending on experimental design. About 14 days after tumour inoculation the mice were sacrificed and the i.p. tumour mass was scored in a blinded fashion on a scale from 0–3. The scoring system was called the peritoneal cancer index (PCI) (Ottow *et al.*, 1987*a*). The mice were eartagged and their numbers recorded prior to scoring. The abdomen of all mice was opened widely and scored for tumour load. A score of 0 is defined as no i.p. tumour, 1 as $\leq 3$ pin point tumour foci (diameter $\leq 1$ mm), 2 as moderate i.p. tumour and 3 as abundant i.p. tumour replacing most of the peritoneal cavity. After all mice were scored the ear tag was read and the data analyzed. In the peritoneal cancer index experiments each experimental group consisting of at least 6 mice were inoculated with $1 \times 10^5$ tumour cells. In the survival experiments mice were inoculated with $1 \times 10^5$ or $3 \times 10^5$ tumour cells. Experimental groups consisted of 12–24 mice.

*Statistical analysis*

Overall significance of difference in the i.p. tumour experiments was examined with the Jonckheere test for trend (Holland & Wolfe, 1973). If this test showed a two sided $P$ value $\leq 0.05$, pairwise comparisons of differences in tumour load were examined with the Wilcoxon rank sum test with a correction for ties (Gehan, 1965). Two-sided $P$-values are presented in all experiments.

### Results

*Tumour reduction by cyclophophamide is dose dependent*

First a dose finding study with CY was performed. BL/6 mice were inoculated i.p. with $1 \times 10^5$ MCA-105 tumour cells. On day 3, 8 or day 3 and 8 different doses of CY, ranging from 10 mg kg$^{-1}$ up to 75 mg kg$^{-1}$ were given i.p. The mice were sacrificed on day 14 and the i.p. tumour load was scored blindly using the PCI scoring system. The results are summarized in Table I. Tumour reduction by CY was clearly dose dependent. In most experiments a significant reduction of i.p. tumour was achieved when doses of 50 mg kg$^{-1}$ or higher were given on day 3. The administration of this dose on day 8 had little if any effect. When doses of 50 mg kg$^{-1}$ or higher were given on day 3 and day 8 a significant tumour reduction was seen in all experiments.

*Chemoimmunotherapy with cyclophosphamide and IL-2 + LAK is superior to either treatment modality alone*

BL/6 mice were inoculated with $1 \times 10^5$ MCA-105 tumour cells. On day 3, or on day 3 and 8, CY (50 mg kg$^{-1}$) was given i.p. On day 3 CY was always given 12 h prior to the administration of IL-2 and LAK cells. IL-2 was administered every 12 h from day 3 through 7 at a dose of 10,000 or 25,000 units depending on experimental design. LAK cells ($1 \times 10^8$) were given i.p. on day 3. Control animals received equal volumes of HBSS as was required for the administration of CY, IL-2 or LAK cells. On day 14 the mice were sacrificed and their i.p. tumour load was scored in a blind fashion.

The results are summarized in Tables II and III.

In Table II the PCI scores are listed concerning the effects of a single administration of CY on day 3 in conjunction with IL-2 and LAK cells.

Table III illustrates the effects after the administration of CY on day 3 and 8 in conjunction with IL-2 and LAK. Treatment with 10,000 units of IL-2 i.p. b.i.d. alone did not reduce i.p. tumour significantly in any experiment, whereas the combination of IL-2 with the adoptive transfer of $1 \times 10^8$ LAK cells i.p. reduced i.p. tumour significantly in all experiments. The tumour reducing effect of a single dose of CY on day 3 was remarkably enhanced by a second dose on day 8, even though a single dose on day 8 had no effect.

**Table I** Effect of different doses of cyclophosphamide on intraperitoneal tumour growth (mean PCI ± s.e.m.)

| *Exp.* | *Day* | *Control* | *Day 3* | *Day 8* | *Days 3–8* |
|---|---|---|---|---|---|
| 1 | 10 mg kg$^{-1}$ | 2.75 ± 0.13 | 2.67 ± 0.21 | 2.50 ± 0.22 | 2.83 ± 0.17 |
| 2 | 25 mg kg$^{-1}$ | 2.75 ± 0.11 | 2.67 ± 0.21 | 2.50 ± 0.34 | 2.17 ± 0.27 |
| 3 | 50 mg kg$^{-1}$ | 2.86 ± 0.10 | 2.00 ± 0.26 (0.004) | – | 1.33 ± 0.33 (0.0003) |
| 4 | 50 mg kg$^{-1}$ | 2.80 ± 0.09 | 2.50 ± 0.22 | 2.83 ± 0.17 | 1.17 ± 0.31 (0.0002) |
| 5 | 75 mg kg$^{-1}$ | 2.75 ± 0.11 | 1.50 ± 0.43 (0.005) | 2.17 ± 0.40 | 0.33 ± 0.14 (0.000005) |

PCI = Peritoneal Cancer Index; s.e.m. = standard error of the mean; cyclophosphamide = CY; $P_2$ values of pairwise comparisons with control animals are indicated between brackets when the PCI of the experimental group was significantly different from the PCI of the control group. The mice were inoculated with $1 \times 10^5$ MCA-105 tumour cells on day 0 and treated with CY at different doses on day 3 or day 8 or on day 3 and 8, as indicated. The mice were sacrificed 14 days after i.p. tumour inoculation and their i.p. tumour load was scored with the PCI-scoring system.

**Table II** Cyclophosphamide on day 3 and IL-2+LAK (mean PCI $\pm$s.e.m.)

| *Exp.* | *Control* | *CY day 3* | *IL-2* | *IL-2/CY* | *IL-2+LAK* | *IL-2+LAK/CY* |
|---|---|---|---|---|---|---|
| 1 | $2.83\pm0.11$ | $2.50\pm0.22$ (NS) | $2.83\pm0.17$ (NS) | $2.33\pm0.21$ $(P_2<0.05)$ | $2.33\pm0.21$ $(P_2<0.05)$ | $2.00\pm0.26$ $(P_2<0.008)$ |
| 2 | $2.87\pm0.09$ | $1.83\pm0.31$ $(P_2<0.002)$ | $2.33\pm0.33$ (NS) | $1.67\pm0.21$ $(P_2<0.0002)$ | $2.00\pm0.26$ $(P_2<0.002)$ | $0.50\pm0.21$ $(P_2<0.00005)$ |
| | | | | | $P_2<0.006$ | |
| | | | | $P_2<0.02$ | | |
| 3 | $2.86\pm0.10$ | $2.09\pm0.23$ $(P_2<0.02)$ | $2.83\pm0.17$ (NS) | $1.83\pm0.17$ $(P_2<0.0005)$ | $1.50\pm0.34$ $(P_20.002)$ | $1.00\pm0.26$ $(P_2<0.0002)$ |
| | | | $P_2<0.007$ | | NS | |
| | | | | $P_2<0.01$ | | |

PCI = Peritoneal Cancer Index; s.e.m. = standard error of the mean; $P_2$-values of pairwise comparisons with control animals are indicated between brackets. $P_2$-values of pairwise comparisons between various treated groups are shown without brackets. Animals were treated with cyclophosphamide (CY) on day 3, 12 h prior to treatment with $1\times10^8$ LAK cells, i.p. on day 3, and/or IL-2, i.p. 10,000 Units, b.i.d., day 3 through 7. Fourteen days after i.p. tumour inoculation the mice were sacrificed and their i.p. tumour load was scored in a blind fashion with the PCI-scoring system.

**Table III** Cyclophosphamide on day 3 and 8 and IL-2+LAK (mean PCI $\pm$s.e.m.)

| *Exp.* | *Control* | *CY 3, 8* | *IL-2* | *IL-2/CY3, 8* | *IL-2+LAK* | *IL-2+LAK/CY3,8* |
|---|---|---|---|---|---|---|
| 1 | $2.83\pm0.11$ | $1.83\pm0.31$ $(P_2<0.006)$ | $2.83\pm0.17$ | $1.83\pm0.31$ $(P_2<0.006)$ | $2.33\pm0.21$ $(P_2<0.05)$ | $1.17\pm0.17$ $(P_2<0.0003)$ |
| | | | $P_2<0.03$ | | $P_2<0.006$ | |
| | | | | $P_2<0.09$ | | |
| 2 | $2.86\pm0.09$ | $0.83\pm0.21$ $(P_2<0.00005)$ | $2.33\pm0.33$ (NS) | $0.33\pm0.21$ $(P_2<0.00005)$ | $2.00\pm0.26$ $(P_2<0.002)$ | $0.0\pm0$ $(P_2<0.00004)$ |
| | | | $P_2<0.005$ | | $P_2<0.002$ | |
| | | | | $P_20.04$ | | |
| 3 | $2.86\pm0.10$ | $1.08\pm0.19$ $(P_2<0.00002)$ | $2.83\pm0.17$ (NS) | $0.50\pm0.21$ $(P_2<0.0002)$ | $1.50\pm0.34$ $(P_20.002)$ | $0.50\pm0.34$ $(P_2<0.0002)$ |
| | | | $P_2<0.003$ | | $P_2<0.06$ | |
| | | | $P_2<0.09$ | | | |
| | | | | (NS) | | |

PCI = Peritoneal Cancer Index; s.e.m. = standard error of the mean; $P_2$-values of pairwise comparisons with control animals are indicated between brackets. $P_2$-values of pairwise comparisons between various treated groups are shown without brackets. Animals were treated with cyclophosphamide (CY) on day 3 and day 8. On day 3 CY was administered 12 h prior to treatment with $1\times10^8$ LAK cells, i.p. on day 3, and/or IL-2, i.p. 10,000 Units, b.i.d., day 3 through 7. Fourteen days after i.p. tumour inoculation the mice were sacrificed and their i.p. tumour load was scored in a blind fashion with the PCI-scoring system.

Combination therapy with IL-2 alone or with IL-2 and LAK cells was more effective than any single component therapy in all experiments. In all experiments tumour reduction was greatest when all three components, e.g., CY, IL-2 and LAK cells were given. In 3 out of 4 experiments no tumour could be detected by the naked eye on day 14 in any or most mice treated with CY with IL-2 or CY with IL-2 and LAK cells.

As shown in Tables II and III, differences in tumour reduction after CY, CY + IL-2, or CY + IL-2 + LAK were not always statistically significant. This is mainly due to the crudeness of the scoring system. The overall trend of the experiments is unmistakably that the more extensive the combined treatment schedule, the more effective its tumour reducing capacity. In order to validate these observations survival experiments were performed.

*Combination treatment of established tumour with CY and IL-2 prolongs survival*

BL/6 mice, inoculated with the usual dose of MCA-105 cell i.p., were treated with intermediate doses of IL-2 (25,000 units, i.p., b.i.d.) and CY ($50\,mg\,kg^{-1}$ on day 3 and 8). A distinct survival benefit was seen with combination treatment (Figure 1). Two mice were long term survivors. After 100 days they were sacrificed and were found to have no visible tumour i.p. upon examination.

*Combination treatment with CY, IL-2 and LAK cells prolongs survival most effectively after high dose tumour inoculation*

BL/6 mice, inoculated with $3\times10^5$ MCA-105 cells i.p. on day 0, were treated with CY ($50\,mg\,kg^{-1}$ on day 3 and 8), and/or IL-2 (25,000 units, i.p., b.i.d., from day 3 through 7) and LAK cells ($1\times10^8$ cells on day 3). IL-2 alone did not prolong survival. The results of this experiment are depicted in Figure 2. Treatment with CY alone, or IL-2 plus LAK cells prolonged survival modestly. A distinct prolongation of survival was seen when CY and IL-2 were combined, even though with this high initial tumour load to permanent survivors were seen. Twenty-five percent of the group of mice that received CY, IL-2 and LAK cells were longtime

**Figure 1** *Significant survival benefit after combination treatment with cyclophosphamide and interleukin-2.* BL/6 mice, inoculated with $1 \times 10^5$ MCA-105 tumour cells i.p. on day 0, were treated with IL-2 (25,000 Units, i.p., b.i.d.) from day 3 through 7 and/or with cyclophosphamide (cytoxan) ($50\,mg\,kg^{-1}$, i.p.) on day 3 and on day 8. $P_2$-values for the significance of pairwise comparisons are indicated in the right upper corner. It is clearly demonstrated that the combination treatment with IL-2 and cytoxan has a synergistic antitumour effect and leads to an important survival benefit.

survivors (no i.p. tumour when autopsy was performed on day 100). Combination treatment with all three components was clearly much more effective than any other treatment schedule.

### Discussion

We have shown that chemoimmunotherapy with cyclophosphamide and IL-2 or IL-2 plus LAK cells has superior antitumour activity when compared to the effects of single agent treatment. The combination of the two treatment modalities has at least an additive if not a synergistic antitumour effect. Low doses of CY in combination with a cycle of intermediate doses of IL-2 resulted in a significant survival benefit and even cured some mice with established i.p. tumour. The treatment of established i.p. tumour by relatively low doses of IL-2 (10,000 units, b.i.d.) alone was not effective but clearly enhanced the antitumour effect of low doses ($50\,mg\,kg^{-1}$) of CY or vice versa. The nature of this phenomenon is unknown but several hypotheses to explain the enhanced antitumour effects may be offered:

(i) First of all the efficacy may be due to the reduction of day 3 tumour load by CY prior to the administration of IL-2. This reduction may be to such a low level of tumour load that treatment with low dose IL-2 will have a further significant tumour reducing effect. It is well known that many biologicals, when used alone, exert significant antitumour effects only when the tumour load is small (Bast & Bast, 1976; Eggermont *et al.*, 1986*a*). It is important to realize that the dose of CY, as used in our experiments, has no negative effect on *in vivo* activation of NK cells (Li *et al.*, 1987).

(ii) The effect of the administration of CY on day 3 may be due to its damage to tumour cells to such a degree that they become more susceptible to lysis by LAK cells, as has been suggested by Papa and coworkers (1988). Conversely immunotherapy may render tumour cells more susceptible to subsequent chemotherapy (Ades *et al.*, 1987), which might explain the efficacy of the administration of a second dose of CY on day 8 in our experiments.

(iii) The significant impact of a second dose of CY on day 8 may well reflect a different mechanism that can potentiate the effects of immunotherapy. Several authors have suggested that a most important mechanism by which CY can enhance immunotherapeutic regimens is by removal of suppressor cells (Polak & Turk, 1974; North, 1982; Greenberg *et al.*, 1985). It has been shown very elegantly by North (1984) that suppressor cells play an increasingly important role seven or more days after tumour inoculation, and that their removal facilitates adoptive immunotherapy.

(iv) Alternatively one may hypothesize that the abrogation of the primary immune response by cyclophosphamide (Anderson *et al.*, 1986; Eggermont *et al.*, 1986*b*) to a weakly immunogenic tumour like MCA-105 removes the cytotoxic T-lymphocytes and thereby an immune cell population that can compete with LAK cells for IL-2. We have demonstrated this phenomenon *in vitro* (Sugarbaker *et al.*, 1987*a*) and reported previously that it can play an important role *in*

**Figure 2** *Combination tretment with CY, IL-2 and LAK cells prolongs survival most effectively after high dose tumour inoculation.* BL/6 mice, inoculated with the high dose of $3 \times 10^5$ MCA-105 tumour cells i.p. on day 0 were treated with $1 \times 10^8$ LAK cells on day 3 and/or with IL-2 (25,000 Units, i.p., b.i.d.) from day 3 through 7 and/or with cyclophosphamide (CY) ($50\,mg\,kg^{-1}$, i.p.) on day 3 and on day 8. $P_2$-values for the significance of pairwise comparisons are indicated in the right upper corner. Combination treatment with CY, IL-2 and LAK cells is clearly superior to any other treatment schedule.

*vivo* in the outcome of treatment with IL-2 and LAK cells (Eggermont *et al.*, 1987*b*).

(v) Finally the hepatotoxic and nephrotoxic effects of IL-2 (Matory *et al.*, 1985) may have altered considerably the pharmacokinetics and metabolism of CY (given on day 8) and may thus have influenced its half life and enhanced its tumour kill.

In conclusion, we have shown that the efficacy of adoptive immunotherapy with low to moderate doses of IL-2 in conjunction with the adoptive transfer of LAK cells can be greatly enhanced by the administration of low doses of cyclophosphamide. This is of importance since the toxicity associated with high doses of IL-2 is quite severe and a major obstacle in obtaining cures with immunotherapeutic regimens alone (Matory *et al.*, 1985; Lotze *et al.*, 1986). In view of the efficacy of a second dose of CY we are currently concentrating our efforts on the development of more effective treatment schedules with repetitive treatment cycles.

### References

ADES, E.W., McKEMIE, III. C.R., WRIGHT, S., PEACOCKE, N., PANTAZIS, C. & LOCKHART, III, W.L. (1987). Chemotherapy subsequent to recombinant interleukin-2 immunotherapy: protocol for enhanced tumoricidal activity. *Nat. Immun. Cell Growth Regul.*, **6**, 260.

ANDERSON, R.E., STAANDEFER, J.C. & TOKUDA, S. (1986). The structural and functional assessment of cytotoxic injury of the immune system with particular reference to the effects of ionizing radiation and cyclophosphamide. *Br. J. Cancer*, **53**, Suppl. VII, 140.

BAST, R.C. & BAST, B.P. (1976). Critical review of previously reported animal studies of tumor immunotherapy and with nonspecific immunostimulants. *Ann. N.Y. Acad. Sci.*, **277**, 60.

DONAHUE, J.H. & ROSENBERG, S.A. (1983). The fate of interleukin-2 after *in vivo* administration. *J. Immunol.*, **130**, 2203.

EGGERMONT, A.M.M., MARQUET, R.L., DE BRUIN, R.W.F. & JEEKEL, J. (1986*a*). Effects of the interferon inducer ABPP on colon cancer in rats; importance of tumor load and tumor site. *Cancer Immunol. Immunother.*, **22**, 217.

EGGERMONT, A.M.M., STELLER, E.P., MATTHEWS, W. & SUGARBAKER, P.H. (1986*b*). Role of the host immune status in the outcome of intraperitoneal immunotherapy with interleukin-2 in mice. *Proc. Am. Assoc. Cancer Res.*, **27**, 342 (*abstract*).

EGGERMONT, A.M.M., STELLER, E.P., OTTOW, R.T., MATTHEWS, W. & SUGARBAKER, P.H. (1987*a*). Augmentation of interleukin-2 immunotherapeutic effects by lymphokine activated killer cells and allogeneic stimulation in murine tumor models. *J. Natl Cancer Inst.*, **79**, 983.

EGGERMONT, A.M.M., STELLER, E.P., MATTHEWS, W. & SUGARBAKER, P.H. (1987*b*). Alloimmune cells consume interleukin-2 and competitively inhibit the anti-tumor effects of lymphokine activated killer cell and interleukin-2 immunotherapy. *Br. J. Cancer*, **56**, 97.

EGGERMONT, A.M.M., SUGARBAKER, P.H., MARQUET, R.L. & JEEKEL, J.J. (1988). *In vivo* generation of lymphokine activated killer cell activity by ABPP and Interleukin-2 and their antitumor effects against immunogenic and nonimmunogenic tumors in murine tumor models. *Cancer Immunol. Immunother.*, **26**, 23.

ETTINGHSAUSEN, S.E., LIPFORD, E.H., MULE, J.J. & ROSENBERG, S.A. (1985). Recombinant interleukin-2 stimulates *in vivo* lymphoid cell proliferation in tissues. *J. Immunol.*, **135**, 1488.

GEHAN, E. (1965). A generalized Wilcoxon test for comparing arbitrarily single sensored samples. *Biometrika*, **52**, 203.

GREENBERG, P.D., KERN, D.E. & CHEEVER, M.A. (1985). Therapy of disseminated leukemia with cyclophosphamide and immune Lyt1+,2−,T− cells. *J. Exp. Med.*, **161**, 1122.

GRIMM, E.A., MAZUMDER, A., ZHANG, H.Z. & ROSENBERG, S.A. (1982). The lymphokine activated killer cell phenomenon: Lysis of NK resistant fresh solid tumor cells by IL-2 autologous activated peripheral blood lymphocytes. *J. Exp. Med.*, **155**, 1823.

HOLLAND, M. & WOLFE, D. (1973). In *Nonparametric Statistical Methods*, p. 144. John Wiley & Sons: New York.

LAFRENIERE, R. & ROSENBERG, S.A. (1985). Successful immunotherapy of experimental hepatic metastases with lymphokine activated killer cells and recombinant interleukin-2. *Cancer Res.*, **45**, 3735.

LI, L.H., DE KONING, T.F. & WALLACE, T.L. (1987). Relationship between modulation of natural killer cell activity and antitumor activity of bropirimine when used in combination with various types of chemotherapeutic drugs. *Cancer Res.*, **47**, 5894.

LOTZE, M.T., MATTORY, Y.L., RAYNER, A.A. & 4 others (1986). Clinical effects and toxicity of interleukin-2 in patients with cancer. *Cancer*, **58**, 2764.

MATORY, Y.L., CHANG, A.E., LIPFORD, E.H., BRAZIEL, R., HYATT, C.L. & ROSENBERG, S.A. (1985). The toxicity of human recombinant interleukin-2 in rats following intravenous infusion. *J. Biol. Resp. Modif.*, **4**, 377.

MULE, J.J., SHU, S., SCHWARTZ, S.L. & ROSENBERG, S.A. (1984). Adoptive immunotherapy of established pulmonary melanoma metastases by the intravenous adoptive transfer of syngeneic lymphocytes activated *in vitro* by interleukin-2. *Science*, **225**, 1487.

MULE, J.J., SHU, S. & ROSENBERG, S.A. (1985). The antitumor efficacy of lymphokine activated killer cells and recombinant interleukin-2 *in vivo*. *J. Immunol.*, **135**, 646.

NORTH, R.J. (1982). Cyclophosphamide facilitated adoptive immunotherapy of an established tumor depends on the elimination of tumor induced suppressor cells. *J. Exp. Med.*, **155**, 1063.

NORTH, R.J. (1984). The murine antitumor response and its therapeutic manipulation. *Adv. Immunol.*, **35**, 89.

OTTOW, R.T., STELLER, E.P., SUGARBAKER, P.H., WESLEY, R.A. & ROSENBERG, S.A. (1987*a*). Immunotherapy of intraperitoneal cancer with interleukin-2 and lymphokine activated killer cells: reduction of tumor and survival benefits in the murine models. *Cell Immunol.*, **104**, 366.

OTTOW, R.T., EGGERMONT, A.M.M., STELLER, E.P., MATTHEWS, W. & SUGARBAKER, P.H. (1987*b*). Requirements for successful immunotherapy of intraperitoneal cancer using interleukin-2 and lymphokine activated killer cells. *Cancer*, **60**, 1465.

PAPA, M.Z., MULE, J.J. & ROSENBERG, S.A. (1986). The antitumor efficacy of lymphokine-activated killer cells and recombinant interleukin-2 *in vivo*: successful immunotherapy of established pulmonary metastases from weakly and nonimmunogenic murine tumors of three distinct histologic types. *Cancer Res.*, **46**, 4973.

PAPA, M.Z., YANG, J.C., VETTO, J.T., SHILONI, E., EISENTHAL, A. & ROSENBERG, S.A. (1988). Combined effects of chemotherapy and interleukin-2 in the therapy of mice with advanced pulmonary tumors. *Cancer Res.*, **48**, 122.

PARKER, G.A. & ROSENBERG, S.A. (1977). Serologic identification of multiple tumor associated antigens on murine sarcomas. *J. Natl Cancer Inst.*, **58**, 1303.

POLAK, L. & TURK, J.L. (1974). Reversal of tolerance by cyclophosphamide through inhibition of suppressor cell activity. *Nature* (Lond.), **249**, 654.

ROSENBERG, S.A., GRIMM, E.A., McGROGAN, M. & 4 others (1984). Biological activity of recombinant human interleukin-2 produced in Escherichia coli. *Science*, **223**, 1412.

ROSENBERG, S.A., LOTZE, M.T., MUUL, L.M. & 10 others (1987). A progress report on the treatment of 157 patients with advanced cancer using lymphokine activated killer cells and interleukin-2. *New Eng. J. Med.*, **316**, 889.

SUGARBAKER, P.H., MATTHEWS, W., STELLER, E.P. & EGGERMONT, A.M.M. (1987). Inhibitory effects of alloimmune T-cells on the cytolytic responses of lymphokine activated killer cells. *J. Biol. Resp. Modif.*, **6**, 430.

WEST, W.H., TAUER, K.W., YANELLI, J.R. & 4 others (1987). Constant-infusion recombinant interleukin-2 adoptive immunotherapy of advanced cancer. *New Engl. J. Med.*, **316**, 898.